# 

**Published:** 1964-10

**Authors:** 


BRISTOL MEDICO-CHURURGICAL JOURNAL
PUBLISHED UNDER THE AUSPICES OF THE

### *Bristol Medico-Chirurgical Society*

The Society was founded in 1874. In 1883 publication of this Journal began, and has continued quarterly since then. During the years 1949 to 1962 it appeared under the title of "The Medical Journal of the South West".

The Society founded a medical library in 1890, which in 1892 was combined with that of the Bristol Medical School. This became the University of Bristol Medical Library in 1925, and an agreement in that year confirmed the privilege of Members to use the University Medical Library.

---

### *Editorial Committee*

Dr. N. J. Brown, Southmead Hospital, Bristol. *Joint Editor.*

Dr. A. B. Raper, Department of Pathology, Bristol Royal Infirmary. *Joint Editor.*

Dr. J. Macrae, Ham Green Hospital.

Dr. R. C. Wofinden, Central Clinic, Bristol.

Mr. A. E. S. Roberts, Medical Library, University of Bristol. *Secretary.*

*Local Correspondents*

*Bath* . . Mr. A. D. Bateman, 3 The Circus, Bath.

*Exeter* . . Dr. L. N. Jackson, Mount Jocelyn, Crediton, Devon.

*Gloucester* . Dr. R. F. Jarrett, 9 College Green, Gloucester.

*Plymouth* . Dr. C. R. Croft, Lexden, Hartley Av., Plymouth.

*Taunton* . Dr. M. McAlley, 1 The Crescent, Taunton.

*Torquay* . Dr. A. Robinson Thomas, 20 Devon Square, Newton Abbot, Devon.

*Truro* . . Dr. F. D. M. Hocking, St. Andrews, Perranporth, Cornwall.

*Yeovil* . . Mr. J. A. Vere Nicoll, Knapp House, Yeovil, Somerset.

### NOTICE TO CONTRIBUTORS

Original articles are invited, provided they have not been published elsewhere. Notes of forthcoming events, meetings held, degrees conferred, staff appointments, and other local news are welcomed. The editorial committee reserves the right to make literary corrections.

Illustrations should always be supplied ready for reproduction. All re-touchings or other operations required will be charged to the author. Line drawings should be drawn in Indian ink. We regret that we must ask authors to pay for making blocks, if this is necessary.

J. W. ARROWSMITH LTD., WINTERSTOKE ROAD
BRISTOL.

### *Examination Results*

University of Bristol

*M.D.:* Monica B. Bishop, S. J. Palmer, M. Le M. Parker, P. C. Sen Gupta, F. G. Spear, M. R. Wills, D. H. Wright.

*Ph.D.* (*Faculty of Medicine*)*:* A. J. Gwinnett, K. V. Mortimer, H. F. Sassoon, Barbara M. Q. Weaver.

*D.P.H.:* N. A. Dent, E. P. Hamblett (*with Distinction*), Gwynneth A. Jones, H. L. Kinman, G. T. Nurse, J. C. Vaccaro, Patricia Vowles.

*M.B., Ch.B.:* A. D. Clark (*Second Class Honours, with Distinction in Obstetrics and Gynaecology*),
Hilary B. Jones (*Second Class Honours, with Distinction in Obstetrics and Gynaecology*),
D. G. Withers (*Second Class Honours*),
B. R. Allen,
Lorna Arblaster,
Pamela M. Ashby,
P. N. Breddy,
K. L. L. Brown,
P. S. Cossham,
Elise L. Davis,
T. J. Deeble,
I. A. R. Dunnett,
A. Edwards,
J. C. W. Evans,
C. M. Fernando,
M. F. Fisher,
D. P. Fossard,
B. A. Gennery,
F. C. Harris,
M. F. Hayes,
D. W. Hillier,
J. Hyde (*with Distinction in Public Health*),
A. S. E. N. Ikedife,
C. W. Jones,
Janice M. Jorgensen,
G. Karmi,
Z. P. Khan,
E. H. Mackay,
C. A. Mathews,
D. J. Moffatt,
R. G. Newhouse,
M. Part,
Maria D. Piorkowska,
J. T. J. Privett,
C. G. L. Raper,
C. Rosse,
Catherine A. Rowntree,
J. A. Sibley,
R. Snook,
D. J. C. Stevens,
I. H. Sutherland,
D. L. Swain,
E. J. Wakley,
M. W. Watson,
J. L. W. Wright,
S. Zabaneh.

### PROGRAMME, 1964–65

| | |
|---|---|
| Wednesday, 14th October: | Annual General Meeting. President-Elect: Dr. G. L. Feneley. |
| Wednesday, 11th November: | Clinical Meeting: Bristol General Hospital. |
| Wednesday, 9th December: | Samuel Wass, M.S., F.R.C.S., Consultant Surgeon to Guy's Hospital: **"Ulcerative Colitis and the Surgeon".** |
| Wednesday, 13th January: | Dr. Donal Early, **"Industrial Rehabilitation".** |
| Wednesday, 10th February: | Mr. R. G. Paul, **"Carcinoma of the Rectum".** |
| Wednesday, 10th March: | Mr. Douglas Phillips, **"Familiar Neurosurgical Problems".** |
| Wednesday, 14th April: | Professor A. I. Darling, **"Health and the Teeth".** |
| Wednesday, 12th May: | Chancellor Garth Moore, Corpus Christi, College, Cambridge, **"The Problem of Death".** |

### INDEX TO VOLUME 79

### I SUBJECTS

Adrenal hormones in carcinoma of bronchus, 6
Ampicillin in enteritis, 119
Amyloidosis, primary, 93
Appointments, 23, 70
Aspergillosis in an okapi, 121
Bath, early medical practice in, 111
Bristol, medical progress in, 103
Bronchus, carcinoma of, 6
Calculus, vesical, 15
Cavers dying of cold, 1
Chlorpropamide in Parkinsonism, 16
Conscious immobility, 35
Examination results, 70, 128
Gerontology, conference on, 68
Herpes zoster, polyneuritis in, 91
Intussusception, 37
Notes and News, 23, 70, 101
Obituaries, 70, 100
Otorhinolaryngology, rise of, 26
Paediatricians' Club, 94
Parkinsonism, 16
Penicillins, trial of, 50
Psychotherapy, 83
Reticulosarcoma, intestinal, 61
Salmonella enteritis, 119
Steatorrhoea, idiopathic, 61
Teenage obstetrics, 115
Transplacental blood loss, 80
Trigeminal nerve, 75
Wessex clinical pathologists, 20

### II AUTHORS

Adams, A. W., 15
Barrington, P. C., 16
Bisson, P. G., 119
Bournes, H. K., 50
Bridges, D., 91
Davis, D. R., 83
Etak, E. S., 91
Fraser, I. D., 80
Hiles, R., 50
James, J. A., 26
Keane, P. M., 6
Lambert, V., 75
Lloyd, O. C., 1
McCarthy, C. F., 6
Marsh, C. A., 111
Mattingly, D., 6
Neale, A. V., 103
Orme, R. l'E., 37
Read, A. E., 6
Rudolf, G. de M., 35
Thomas, W. R. G., 50
Wingate, M. B., 115
Woodyard, J., 50

*Printed by*
J. W. Arrowsmith Ltd., Bristol

